# The Treatment of Retinal Detachments by the Injection of Iodine Tincture into the Vitreous Chamber

**Published:** 1890-06

**Authors:** S. Latimer Phillips

**Affiliations:** Savannah, Ga.


### THE TREATMENT OF RETINAL DETACHMENTS BY THE INJECTON OF IODINE TINCTURE INTO THE VITREOUS CHAMBER.

BY S. LATIMER PHILLIPS, M. D., OF SAVANNAH, GA.

Oculists recognize retinal detachment as one of the most formidable pathological conditions presented to them, and thus any remedy brought forth to alleviate this is most gladly received.

Recently, Prof. Schœler,* of Berlin, has brought forward a means of cure for this trouble which promises to be of the greatest importance. It is that it may be more generally known that I wish to call attention to it. When referring to the treatment of this condition most authors speak more or less discouragingly.

A number of these cases are caused by traumatism, blood or serum being effused between the choroid and retina. Many others are the result of progressive myopia, there being a pulling away of the sclerotic from the retina in elongation of the eye-ball, posteriorly, or a special tendency of the anterior surface of the choroid to serous effusion. For others there is no known cause.

Leber holds that the immediate cause is always a tear in the retina. This is at times almost impossible to be found. The vitreous body, shrinking away from the retina, brings the latter with it, where it is most firmly attached, and thus causing it to give away at that point. Through this opening the liquid transudes. Schœler is of the opinion that from 80 to 90 per cent. of retinal detachments are produced in this manner, and it is upon this that he has based his method of treatment.

Varied has been the means of treating retinal detachments, both by medicine and surgery. Patients have been put to bed on their backs in a darkened room and required to stay there for a certain length of time. Some have had medicine and others have not ; some have improved, a few have been cured, but many have

*Zur Operativen Behandlung und Heilung der Netzhautablosung, Berlin, 1889.

had no benefit at all. Antiphlogistics and derivatives have been employed. Hypodermics of pilocarpin, jaborandi per mouth, mercurial inunction, artificial leech, each had its advocate.

I believe Sichel, of Paris, was the first to attempt to remove the effusion, but without very good results. Great care was taken in puncturing the sclera and choroid not to go through the retina, as it was thought to make the condition worse. Von Græfe sought afterwards to arrest the progress of the disease by establishing communication between the pouch back of the retina and the vitreous body. Bowman also did an operation somewhat similar. DeWecker has proposed to keep up continuous drainage by putting a gold thread through sclerotic and choroid. He has also perforated these two membranes with the galvanic cautery, but these operatives have been of no special good. Galezowski devised a little pump aspirator after that of Dieulafoy for removing the accumulated fluid. He was the first to practice *iridectomy** and obtain good results in retinal detachment. All of the above surgical procedures have done more or less good, but the chances are that, in many cases, more harm than good has resulted. In making observations upon rabbits' eyes of injections of various antiseptics to test their value in septic choroiditis, Schœler had results which were such as to make him think that this treatment might be of use in retinal detachments. After sundry experiments with a number of drugs, tincture of iodine was finally settled upon as the only preparation of any real use in this trouble. It was sufficiently strong to do the work and not too irritating. This was injected through the sclerotic, choroid and retina into the vitreous chamber, in preference above the detachment and near the supposed opening. This was done with a hypodermic syringe but with a curved instead of a straight point, and from 2 to 6 drops injected. The vitreous is compressed, the retina shoved towards the choroid, and any old adhesions torn up. The fluid, now diluted, passing through the rent, comes in contact with the other surface of the retina, producing adhesive inflammation ; the retina being then in contact with the choroid becomes firmly attached. The earlier these injections are under-

*Galezowski, Traite des Maladies des Yeux, p. 657, Paris, 1888.

taken the better ; for when the retina becomes wholly detached from the choroid, being fast only at the optic nerve and ora serrata, or when degenerative changes have taken place, it is too late for any treatment. Of course, after this operation, patient is put to bed, with compress bandage over eyes, and kept there one or two weeks. Under these circumstances a light or milk diet is preferable. If there is much pain, which is likely, morphia, hypodermically, can be given. The process of healing is watched by the use of the ophthalmoscope.

Schœler* reports five cases in detail. In these five cases the results have been most satisfactory, and if the results of others prove equally so, indeed, a new epoch in ophthalmology will have been inaugurated.

